# Implementation of a strategy to facilitate effective medical follow-up for Australian First Nations children hospitalised with lower respiratory tract infections: study protocol

**DOI:** 10.1186/s12890-022-01878-3

**Published:** 2022-03-17

**Authors:** André Schultz, Anne B. Chang, Fenella Gill, Roz Walker, Melanie Barwick, Sarah Munns, Matthew N. Cooper, Richard Norman, Pamela Laird

**Affiliations:** 1 Wal-yan Respiratory Research Centre, Telethon Kids Institute, Perth, WA Australia; 2 Department of Respiratory and Sleep Medicine, Perth Children’s Hospital, Perth, WA Australia; 3 Department of Paediatrics, School of Medicine, University of WA, Perth, Australia; 4 Child Health Division Menzies School of Health Research, Darwin, NT Australia; 5 Department of Respiratory Medicine, Queensland Children’s Hospital, Brisbane, QLD Australia; 6 Australian Centre For Health Services Innovation, Qld University of Technology, Brisbane, QLD Australia; 7 Nursing Research, Perth Children’s Hospital, Perth, WA Australia; 8 School of Nursing, Faculty of Health Sciences, Curtin University, Bentley, WA Australia; 9 School of Indigenous Studies, Poche Centre for Indigenous Health, University of Western Australia, Perth, WA Australia; 10 School of Population Health, University of Western Australia, Perth, WA Australia; 11 Ngangk Yira Institute for Change, Murdoch University, Perth, WA Australia; 12 Hospital for Sick Children, Toronto, Canada; 13 Department of Psychiatry, Temerty Faculty of Medicine, University of Toronto, Toronto, Canada; 14 Dalla Lana School of Public Health, University of Toronto, Toronto, Canada; 15 Telethon Kids Institute, University of Western Australia, Perth, WA Australia; 16 School of Population Health, Curtin University, Bentley, WA Australia; 17 Department Physiotherapy, Perth Children’s Hospital, Perth, WA Australia

**Keywords:** First Nations children, Knowledge translation, Chest infections

## Abstract

**Background:**

First Nations children hospitalised with acute lower respiratory infections (ALRIs) are at increased risk of future bronchiectasis (up to 15–19%) within 24-months post-hospitalisation. An identified predictive factor is persistent wet cough a month after hospitalisation and this is likely related to protracted bacterial bronchitis which can progress to bronchiectasis, if untreated. Thus, screening for, and optimally managing, persistent wet cough one-month post-hospitalisation potentially prevents bronchiectasis in First Nations’ children. Our study aims to improve the post-hospitalisation medical follow-up for First Nations children hospitalised with ALRIs and thus lead to improved respiratory health. We hypothesize that implementation of a strategy, conducted in a culturally secure manner, that is informed by barriers and facilitators identified by both parents and health care providers, will improve medical follow-up and management of First Nations children hospitalized with ALRIs.

**Methods:**

Our trial is a multi-centre, pseudo-randomized stepped wedge design where the implementation of the strategy is tailored for each study site through a combined Participatory Action Research and implementation science approach informed by the Consolidated Framework of Implementation Research. Outcome measures will consist of three categories related to (i) health, (ii) economics and (iii) implementation. The primary outcome measure will be Cough-specific Quality of Life (PC-QoL). Outcomes will be measures at each study site/cluster in three different stages i.e., (i) nil-intervention control group, (ii) health information only control group and (iii) post-intervention group.

**Discussion:**

If our hypothesis is correct, our study findings will translate to improved health outcomes (cough related quality of life) in children who have persistent wet cough a month after hospitalization for an ALRI.

*Trial registration* ACTRN12622000224729, prospectively registered 8 February 2022, URL: https://www.anzctr.org.au/Trial/Registration/TrialReview.aspx?id=382886&isReview=true.

**Supplementary Information:**

The online version contains supplementary material available at 10.1186/s12890-022-01878-3.

## Background

Respiratory illnesses account for 12% of the age-standardised mortality gap between First Nations and other Australians [1]. Respiratory disease is the second most common cause for hospitalisation among First Nations people [2]. First Nations children hospitalised with acute lower respiratory infections (ALRIs) are particularly vulnerable to developing bronchiectasis [3, 4], with 15–19% of First Nations children having bronchiectasis within 24-months post-hospitalisation with pneumonia or bronchiolitis [3, 4]. Children who present with persistent wet cough a month after hospitalisation are specifically at risk because they likely have protracted bacterial bronchitis which can progress to bronchiectasis [5]. If children are screened for persistent wet cough one-month post-hospitalisation and managed optimally if wet cough is still present, then bronchiectasis can potentially be prevented. Culturally secure assessment [6] and treatment [7–9] is important for optimal outcomes in First Nations contexts. That is, ensuring First Nations Australians receive care that acknowledges their unique cultural needs and differences.

The study protocol follows the Standard Protocol Items: Recommendations for Intervention Trials [10] (SPIRIT) and reporting will follow the Standards for Reporting Implementation Research (STaRI) [11]. The Consolidated Criteria for Reporting Qualitative research [12] (COREQ) will be used to report qualitative data findings.

### Overall aim

To facilitate timely and effective post-hospitalisation follow-up of First Nations children hospitalised with ALRIs to optimise respiratory health.

### Specific objectives

To implement an effective medical follow-up strategy that will:

1. Improve post-hospitalisation follow-up rates;
2. Improve the quality of follow-up for First Nations children hospitalised with ALRIs;
3. Improve the cough-related quality of life of First Nations children who have persistent wet cough one-month post-hospitalisation for ALRI;
4. Determine the health-related economics of the strategy.
5. Determine the best implementation practice for scaling up of the strategy.

## Methods

### Trial design

**Fig. 1:**
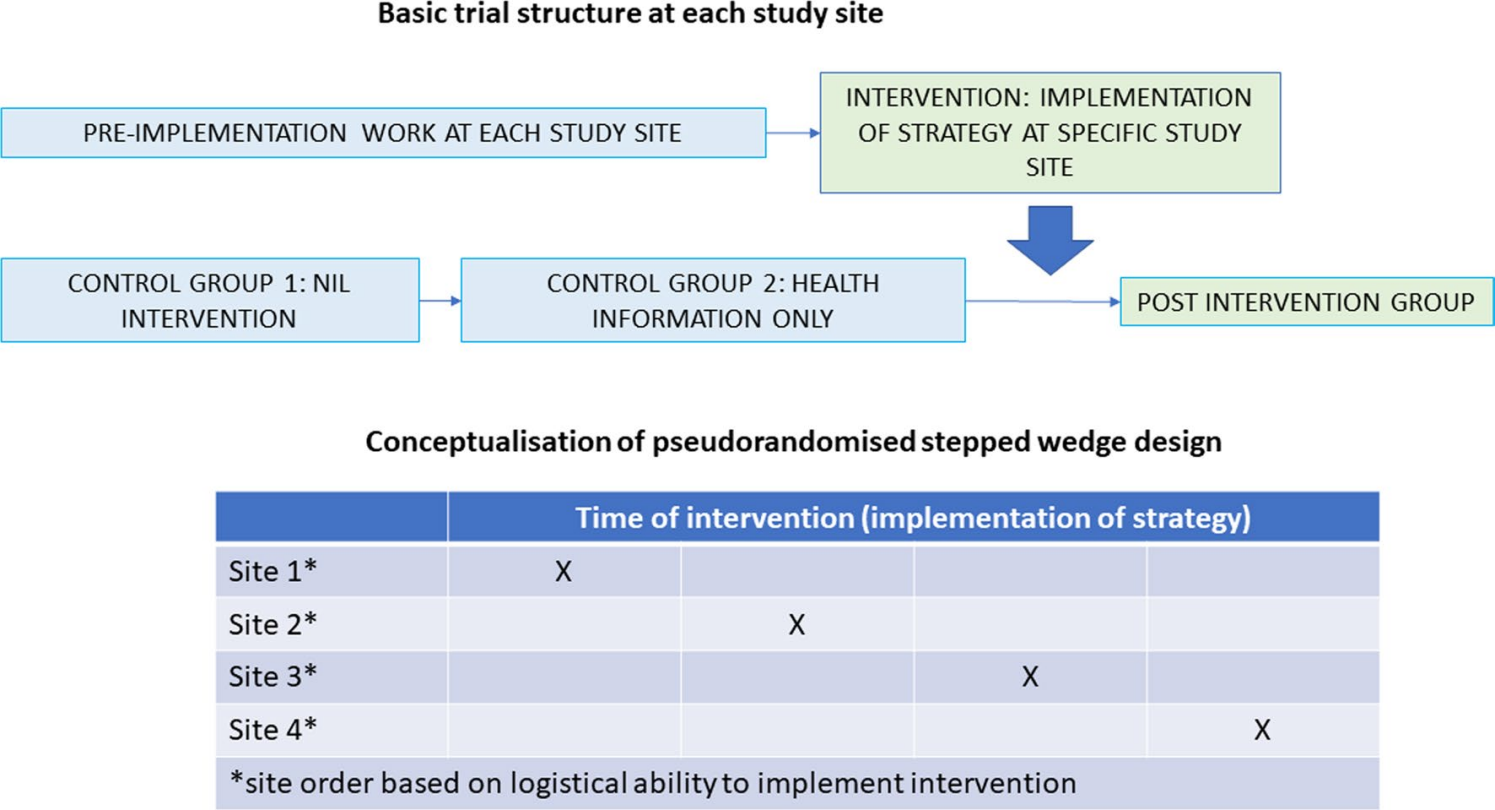
Overview of trial design

The study is a multi-centre, pseudo-randomized stepped wedge design where the medical follow-up strategy at each study site is implemented with consideration of barriers and facilitators using a combined Participatory Action Research (PAR) and implementation science approach, guided by the Consolidated Framework of Implementation Research. Outcome data for each site will be collected in three stages, as discussed below. The timing of interventions at individual study sites/cluster will not be randomised but determined by site-related logistics which will vary greatly (months to years) between sites (Fig. 1).

### Participants, interventions and outcomes

#### Study setting

Settings include at least two Australian hospitals with an associated primary care health service that provides medical care to primarily First Nations.

Sites:

1. Broome: Broome Hospital, Broome Regional Aboriginal Medical Service; Kimberley Aboriginal Medical Service Broome, Western Australia
2. Port Hedland Health Campus, Wirraka Maya Aboriginal Medical Health Service Western Australia

We hope to extend the study to additional sites, pending funding. These additional sites are:

3. Karratha Health Campus and Mawarnkarra Health Service
4. Townsville hospital and local First Nations health service
5. Cairns hospital and local First Nations health service

### Eligibility criteria

#### Participants

**Inclusion criteria:** Australian First Nations children aged 0–16 years hospitalised with ALRIs and their parents/carers (henceforth, respectfully called parents) who will serve as proxy for their children.

Three participant groups:

1. **Nil-intervention control group**: Children hospitalised with ALRIs between 6-weeks to 6-months before commencement of our trial.
2. **Health-information control group**: Prospectively recruited children hospitalised with ALRIs after commencement of the trial but before the implementation has commenced.
3. **Post-intervention group**: Children hospitalised with ALRIs after the implementation of the strategy has commenced (from Day-1 of the implementation of strategy).

#### Participant exclusion criteria

- Non-First Nations children.
- Children over the age of 16 years.
- Children who have a diagnosis of cystic fibrosis.
- Children with a tracheostomy insitu or have been admitted to hospital to undergo this procedure.

### Who will take informed consent?

A trained research officer will contact potential participants/parents and undertake the informed consent process. This person will use culturally secure patient information documents that have undergone a rigorous development process that included feedback from stakeholders and 18 Western Australian First Nations community members from regional, remote and metropolitan areas.

### Additional consent provisions for collection and use of participant data and biological specimens

Confidentiality of enrolled participants will be ensured through use of a unique identifier code for all collected, shared, and maintained data to protect confidentiality before, during, and after the study. De-identified data for analysis will be stored in REDCap software (secured, passcode protected and encrypted research database).

Participants can withdraw consent at any time and do not need to provide a reason. If a participant wishes to withdraw from the study, the process for withdrawal is outlined on the consent form. Once withdrawal of consent has been obtained, no further data will be collected. Participants will be informed that their clinical care will not be affected if they choose to withdraw at any time.

### Interventions

#### Explanation for the choice of comparators

Each site will have three groups of participants, (i) Nil intervention control group, (ii) Health information only control group, and post-intervention group.

#### Intervention description

The medical follow-up strategy (henceforth termed ‘strategy’) was informed by our previous research [13, 14] and methodological approaches [15], and is consistent with Australian [16] and International [17] guidelines for children at high risk of developing bronchiectasis. Implementation of the strategy core components will be adapted to the unique context of each study site; the core components are outlined in Table 1.

**Table 1:** Core components of medical follow-up strategy

Core components of the medical follow-up strategy	How each component will be provided?	Who will be involved in providing/delivering the core component?	Outputs	Outcomes (How we will know the core component was delivered as intended)
1. First Nations lead	• Advisory lead on all cultural components of project	• First Nations lead	• Culturally secure operations with First Nations knowledge privileged	• First Nations lead identified and appointed and employed for duration of study
2. Stakeholder engagement	• Engagement occurs with focus groups and interviews for the purpose of identifying current state, barriers and facilitators to providing the strategy • Inner setting: patients, HCPs, executive • Outer setting: primary care HCPs and other key stakeholders such as umbrella First Nations primary care organisation) and the Telethon Kids BREATH team consumer reference group	• Qualitative experts will lead the interviews, focus groups and analysis • Interviews and focus groups will capture voice of parents, HCPs and primary care HCPs involved with children hospitalised with ALRIs	• Identified barriers and facilitators	• Written report summarizing key findings, process map and tailored implementation of strategy for each site
3. Training of clinicians and other health staff	• Online module and podcast(s) accessible and free of charge • In-person training	• Clinical champion will promote module and podcast at each site • In-person clinical training by pediatric respiratory clinician • Cultural training by Aboriginal cultural educator	• Module and podcast hosted on National website • Clinical/cultural training accomplished	• Metrics of completions at each site (electronic capture system) • Percentage or proportion of HCPs trained out of the total number (denominator) who could be trained at each site
4. Educational resources	1. Health information flip chart 2. Health facts sheet for parents 3. Letter to local clinic	• Champion at each site ensures accessibility of documents	• Educational resources adapted for each site • Available on internet/intranet • Hard copies easily accessible in clinical workrooms	
5. Patient admission process	1. Patient is identified as being First Nations and local clinic is updated on electronic record system 2. Parent receives lung health education and is instructed to follow-up in 1-month at local clinic	1. Clerks 2. Doctors 3. Nurses	• Automated SMS sent to parent	• Audit of discharge summary to check First Nations identification and local clinic contact
6. SMS follow-up reminder	• A SMS is sent to each patient at 4-weeks post discharge with reminder to take child to local clinic for follow-up if chronic wet cough	• Site Information Technology (IT) department	• Automated SMS sent to each patient at 4-weeks post discharge	• Number of SMS reminders recorded
7. Discharge process	• Electronic discharge summary requires instructions for primary care clinicians • Instructions to be integrated into the existing electronic discharge form via template	• Doctor to complete discharge summary with all required information • IT technician at hospital site to embed template with instructions for easy integration by doctor when completing summary	• Instruction template is integrated into the electronic discharge summary system	• Audit of discharge summary compliance with required information (see Additional file 6)
8. Local champions	• Champion helps HCPs to uptake new processes and reminds HCPs about best practice at routine staff meetings	• Identified by clinical lead at each site	• Champion is provided with training at each site	• Number of champions and interactions recorded at each site

*HCP* healthcare provider, *IT* information technology, *SMS* short message service

### Criteria for discontinuing or modifying allocated interventions

The medical follow-up strategy was developed in a study at Perth Children’s Hospital [14]. Delivery of the strategy’s core components (Table 1) will be adapted to the workflows and structure of sites using a combined decolonising research approach [15], which includes Participatory Action Research (PAR) [14] and the Consolidated Framework for Implementation Research (CFIR). The CFIR provides a structure for identifying a range of factors that can facilitate or hinder the implementation of evidence-informed interventions. This implementation approach aims to maximize translation potential through the continuous process of action, reflection and feedback allowing adjustments to the project throughout all phases [18]. In this way, the implementation can be optimised to ensure the benefits of the strategy are sustainable over the long term, beyond the project, and that scale-up is feasible across other service provision sites. Implementation science informs our approach, alongside context sensitive adjustments of implementation processes that consider First Nations consumer views to ensure engagement, effectiveness, and sustainability. Identification of adaptations required for implementation of strategy core components will involve interviews and focus groups with stakeholders, including:

1. Healthcare providers (HCP): Purposive sampling to recruit HCPs at hospital or local primary care clinic who are involved in caring for patients or providing follow-up following an admission for ALRI.
2. Parents: Purposive sampling to recruit parents of First Nations children hospitalised with ALRI. A First Nations researcher will be available for all interviews with First Nations participants.

Inclusion criteria of participants for interviews:

1. HCP: Any HCP within the hospital or local clinic who are involved in the medical care of First Nations children with ALRI, including but not limited to doctors (paediatricians, respiratory physicians, service coordinators, Aboriginal health practitioners, primary-care liaison personnel), nurses and clerks.
2. Parents of First Nations children hospitalised with ALRI. Parents may include grandparents, foster parents or other family members within the kinship system, who are responsible for caring for the hospitalised child.
3. External stakeholders: Relevant stakeholders such as the Aboriginal Community Controlled Health Councils at a state and local level, peak bodies, such as the Aboriginal Health Council of Western Australia.

The purpose of the interviews and focus groups is to ascertain barriers and facilitators to implementing the strategy and to understand the perspectives of parents and HCPs about the need for medical follow-up. Interviews and focus groups will be conducted by at least two interviewers: a content expert (clinician or Aboriginal lead) and a qualitative researcher. Interview participants will be recruited during the “health information only” period, prior to the implementation of the strategy.

Interviews and focus groups will be recorded by audio or with hand-written notes by two researchers (depending on interviewee preference). The interview guides (see Additional file 1, Additional file 2, Additional file 3) were developed by two researchers with experience in qualitative methods and a pediatric respiratory physician. The draft questions were tested with two families and two HCPs at Perth Children’s Hospital and then refined. Audio taped interviews will be transcribed verbatim and notes compared and checked with at least two qualitative research experts. Issues related to confidentiality, interview bias, role designation, power imbalance and assumptions influencing participants will be discussed and resolved within the research team. Interviewee transcript review will be undertaken when clarification is necessary and/or if the interviewee has indicated they would like to review their transcript.

Qualitative component sample size (interviews and focus groups)

1. HCPs: ~ 8–10 focus groups with 6–8 participants and ~ 10 individual interviews per site.
2. Parents: ~ 15–20 individual interviews per site.

Sample size for the qualitative component was estimated considering the concept of information power [19] and the impacting dimensions; study aim, sample specificity, use of established theory, anticipated quality of dialogue and the analysis strategy. Participants will be recruited to the parent and HCP groups respectively until thematic saturation is achieved for each group i.e., no new information or themes are evident.

#### Analysis of qualitative data (interviews and focus groups)

Reflexive thematic analysis, using deductive and inductive analysis [20] informed by Creswell and Creswell’s six-steps [21] will be undertaken to map themes by identifying codes and then sub-themes as agreed by the researchers using an iterative process. Implementation factors (CFIR) identified in the interviews will inform implementation processes (e.g., strategies for mitigating barriers) at each site. Trustworthiness will be achieved by ensuring the researchers address four key criteria outlined by Nowell et al. [22]. The Consolidated Criteria for Reporting Qualitative research [12] (COREQ) will be used to report focus groups and interview data.

### Strategies to improve adherence to interventions

Fidelity to the strategy at each site will be managed through tracking log of outputs and with field notes. Implementation process will be tracked with fieldnotes.

**Relevant concomitant care permitted or prohibited during the trial**

N/A

**Provisions for post-trial care**

N/A

### Outcomes

The study outcomes consist of three categories: (i) health, (ii) economics and (iii) implementation.

### Health outcome/primary outcome measure (Quality of Life (PC-QoL))

The primary outcome is the parent-proxy Cough-specific Quality of Life (PC-QoL) for children with chronic wet cough. Effective follow-up and management of children hospitalized with ALRIs will be assessed by measuring PC-QoL between 6 weeks and 3 months after hospitalization. We will use the 8-item version of the PC-QOL tool that has been shown to be a valid, reliable, and responsive tool for measuring the burden of chronic cough in children. This tool has been used effectively in First Nations settings [17, 18].

Rationale: children with ongoing respiratory symptoms and cough post hospitalization will have improved quality of life if they receive effective medical follow-up at one-month post discharge. The PC-QoL scores will be compared pre and post implementation in different children who had a chronic wet cough post-discharge. Children with chronic wet cough post-hospitalization who received timely follow-up and optimal management will have better cough specific QoL than children who did not. It has been determined, using the anchor method, that 0.9 is the calculated minimum important difference in PC-QOL [23].

Respiratory symptoms after ALRI should typically resolve within 4 weeks. Children with ongoing wet cough 4 weeks post-hospitalization are likely to have protracted bacterial bronchitis that should resolve if detected and managed in a timely way. Hence, if children receive appropriate follow-up and management a month after discharge then we expect their cough related QoL to be better after 6–12 weeks than those who did not (waiting 6–12 weeks allows time for management to be completed in those children with chronic wet cough who may require up to a month of antibiotic treatment).

The PC-QoL tool will be administered by a research officer in-person at the hospital sites, over the phone, or electronically (online option via REDCap for those participants who would prefer this option). Participants will be contacted by the research officer via text message or telephone.

### Secondary outcome measures

1. Cultural competency of HCP while child hospitalized (parent report).
2. Instructions for medical follow up given by clinician (parent report).
3. Follow-up rates of children within 1-month (range 3–6 weeks) post hospitalization for ALRI (parent report).
4. In children with chronic wet cough:
  - Antibiotics prescribed at follow-up (parent report).
  - Adherence to prescribed antibiotics (self-report).
  - Cough resolution at 6–12 weeks post discharge (parent report).
5. Discharge summary audit: An audit of the discharge summary to evaluate patient management (see Additional file 6):
  - First Nations ethnicity recorded.
  - Local clinic/doctor identified.
  - Instructions given to parent to follow up in 1-month with local doctor.
  - Instructions to the local clinic/GP clearly stipulates to check child for ongoing chronic wet cough, absence or presence of ongoing other signs and suggest appropriate management if chronic wet cough is present. Link to appropriate clinical practice guidelines for the management of chronic wet cough provided.
    *Note: an audit of discharge summaries will be conducted each month. De-identified results (audit and feedback) will be provided to the HCPs each month at their regular departmental meeting to facilitate service improvement as part of implementation process.*

Secondary outcome measures 1–4 will be captured by telephone follow-up at 6–12 weeks post discharge. To ensure culturally secure engagement, a standardized script will be used to provide context by the telephoning research officer. The research officer who telephones the parent will first introduce themself and identify from where they are calling. The officer will provide context for the call, i.e., remind parent about the study and child’s recruitment at recent hospital admission. The questions are outlined in question 2, 3, 5 of Additional file 7.

### Economic analysis

A stepped health economic evaluation will be undertaken to determine the cost-effectiveness of implementation and the economic cost-saving of cases with prevented bronchiectasis. Costs determined are: (i) Cost of provision of new service, including staffing, consumables, cost of training, electronic discharge system modifications; (ii) the extra cost of caring for a child with bronchiectasis over their lifetime for ~ 19% of children hospitalised with ALRI.

The economic evaluation aims to determine the cost of the implementation process, i.e., the cost of the medical follow-up strategy at a hospital and primary care clinic and to determine the cost-effectiveness of the program though evaluating the cost-saving from preventing bronchiectasis in children i.e., where a child who had chronic wet cough and received appropriate follow-up and management thereby resulting in resolution of the cough will be seen as a case of bronchiectasis prevented. Cost data for the economic evaluation will include all costs associated with implementation at the hospital and primary care clinic (i.e., the strategy cost) and will include costs such as training workshops, health organization changes in process or functions at both hospital and local primary care clinic, facilitation of staff behaviour change, including identification of champions, audit and feedback, identification of barriers and enablers, and ongoing engagement with facilitation of practice change. The cost of the implementation strategy will be based on the current implementation package at Perth Children’s Hospital (PCH), adapted for other participating hospitals. This is the best estimate based on data, thus far, to deliver this strategy at a hospital site.

The economic evaluation will present findings as a series of steps, first reporting the costs and outcomes of the strategy separately, then reporting a series of incremental cost-effectiveness ratios (ICERs) using a variety of key outcomes, including the number of children managed, and the estimated number of cases of bronchiectasis prevented.

### Implementation outcomes

The following implementation outcomes will be measured to evaluate implementation success for the strategy [24]:

- Fidelity to core components of the strategy will be assessed as per Table 1 outcomes.
- Acceptability (“the perception among implementation stakeholders that the strategy is agreeable… and satisfactory” [24]) will be assessed for HCPs and parents through semi-structured interviews (see Additional file 1, Additional file 2, Additional file 3, Additional file 4, Additional file 5).
- Appropriateness (“the perceived fit of the strategy for the given practice setting” [24]) will be assessed with the 22-item TCU Workshop Evaluation (WEVAL) form to assess the perceptions of the strategy directly after implementation (http://ibr.tcu.edu/wp-content/uploads/2013/06/weval.pdf) and the 14-item TCU Workshop Assessment Follow-Up (WAFU) to assess changes 6-months after (http://ibr.tcu.edu/wp-content/uploads/2013/06/wafu.pdf).
- Implementation cost will be addressed as described under the economic analysis section.
- Penetration and adoption will be assessed as (i) the number of HCPs who participated in training out of the total number of HCPs, and (ii) audit of discharge summaries as described in secondary outcome measures.
- Sustainability will be addressed through focus groups and interviews with HCPs and parents in the final months of the project and feedback questionnaires for HCPs.

We acknowledge that given the study is being conducted in First Nations settings, evaluation of implementation outcomes must consider the diversity within First Nations contexts [25]. Therefore, assessment of “acceptability” and “appropriateness” will include identification of First Nations leadership, governance, co-design, partnership and involvement in implementation processes.

### Study and participant timeline

The timeline for the study is outlined in Table 2.

**Table 2:**
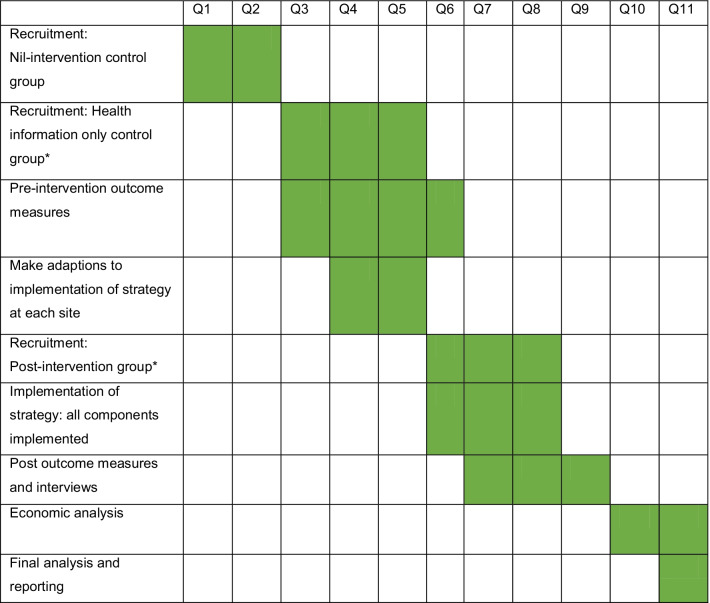
Timeline of study for ALL sites*

Timing of Q1 at each site/cluster will vary. The table above is a conceptual map showing the chronology only. Time intervals between different stages will vary due to local site-related logistics

*Q* quarter

^*^The above timeline applies to individual study sites/clusters

### Sample size

The primary outcome measure for this trial is the PC-QOL (8-item version). There is limited data available to inform a robust sample size calculation and it is acknowledged that there are challenges with estimating potential cohort sizes within the limited remote communities included in this study, thus a pragmatic approach has been taken to the sample size calculations. Data (unpublished) from our previous studies using the PC-QOL suggests a conservative standard deviation of 1.3, a historic mean score of approximately 2.7, a pre-implementation mean score of approximately 3.6, and a post-implementation mean score of approximately 4.3. Two calculations were carried out, one based on a two-sided T-Test (to reflect an examination of the intervention effect within a single site), and the other based on the overall analysis (reflecting the clustered, within site, nature of the data).

Single site analysis with at least 12 participants with chronic wet cough in each period will have > 80% power to detect a nil-intervention (2.7) v post-intervention (4.3) difference of 1.6, alpha 0.05. Full sample (clustered) analysis with at least 5 participants per cluster (4) per period (assuming COV 0.65, ICC 0.01) will have > 95% power to detect a nil-intervention (2.7) v post-intervention (4.3) difference of 1.6, alpha 0.05; at least 10 participants per cluster (4) per period will be required to have > 80% power to detect a difference of 0.9 (alpha 0.05). Adjustments for multiple comparisons were not made as calculations are to provide guidance only, with final sample size being dependent on cohort sizes and diagnosis counts during observation periods; sample size calculations were carried out using PASS [26].

#### Pragmatic data collection

Ultimately, the studies power will depend on the ability to recruit participants from the limited (and difficult to estimate) pool of diagnosed patients within the study population. We estimate that within each of the three stages, somewhere between 50 to 150 eligible children will be recruited (based on an examination of historic data from the study sites). Of these, we expect ~ 20% to have chronic wet cough and meet the eligibility criteria to have their PC-QoL assessed. Thus, we aim to have data for 20 participants per combined regional sites per stage. Prior data, coupled with the above sample size calculation, determines the feasibility of the study.

### Recruitment

#### Recruitment of Nil-intervention control group

A researcher will access hospital records of patients who have been previously (within the last 6-months) hospitalised at the hospital site with ALRIs. A recruitment letter will be sent to the parent from the hospital containing an overview of the study and opt-out instructions. If an opt-out response was not received within 3 weeks of posting the letter, a clinician researcher will contact the parent via telephone. The parent will be given the opportunity to consent via telephone. The questions will be administered via telephone.

#### Recruitment of participants on hospital wards

**In General:**

Health-information control group and post-intervention group:

1. Identified through hospital electronic record systems or equivalent to identify if a First Nations child has been admitted with an ALRI.
2. A researcher will invite the parent, while on the ward ***during the child’s admission***, to participate in the study using the following process:
  - Lung health information will be given to parents in a culturally secure way, which includes the use of an information flip chart [27].
  - The study will be explained verbally and via written study information.
  - Parents will be invited to participate in the study (with follow-up) and to consent or opt out of an interview.
  - If consenting, an interview will be conducted.
  - A First Nations researcher will be available for all interactions with First Nations participants.
  - During recruitment, parents will be asked for the best contact number for a follow-up phone call to obtain follow-up data from parents.

#### Recruitment during COVID restrictions

1. Researcher will access admission records to identify if a First Nations child has been admitted with an ALRI (current protocol).
2. A culturally secure information pack complete with an information pamphlet (see attached information pamphlet_COVID-19_ V3-03042020) explaining lung health and acute and chronic chest infections, a brief explanation letter to parents (see Cover Letter for Families_COVID-19_V3_03042020), the study information sheet and consent form will be left at the door outside patient rooms in a clearly marked folder/envelope.
3. The researcher will send a text message (attached Recruitment Text_COVID-19_V1_190320) to the patient’s parent. The text message will have a poster attachment (see attached Recruitment poster_COVID-19_V1_19032020) and (if recipient does not use a smartphone) a short blurb about the study to invite the parent to participate or return text to “opt out”.
4. The researcher will telephone the parent after at least 30 min if no “opt-out” text is returned to provide information and go through the informed consent process. The 30-min opt-out window considers that many patients are admitted overnight only for acute respiratory conditions and are discharged from hospital in the morning after an overnight stay. Thus, the timeframe for recruitment is limited.
5. Parent may give verbal consent or choose to fill out the paper copy they received. The paper copy will be retrieved by the research team.

### Assignment of interventions: allocation

**Sequence generation**

N/A

**Concealment mechanism**

N/A

**Implementation**

N/A

**Assignment of interventions: Blinding**

**Who will be blinded**

N/A

**Procedure for unblinding if needed**

N/A

### Plans for assessment and collection of outcomes

A PC-QoL tool [23] will be administered to eligible participants (not attached here in for copyright reasons, but for tool provided). The PC-QoL tool [23] has eight questions rated on a Likert-scale, and administered using REDCap software. A researcher who is trained and skilled in REDCAP software will create, maintain and enter data onto the database for all sites.

Participants will have their unique medical record unit number attached to their data, so will be re-identifiable. The use of this unique identifier will also allow us to link multiple records for the same participant together, as it is anticipated that a small (but non-negligible) number of participants will have recurrent chronic wet cough and hence be likely to be captured on two or more occasions spanning different data collection periods. The database will be stored on a computer at a location requiring an access card swipe for access. The computer is accessed by a password lock. Participants’ information will only be used for the purposes of this research project and will only be disclosed with permission except where required by law.

It is anticipated that the results of this research project will be published and/or presented in a variety of fora. In any publication and/or presentation, information will be provided in such a way that a participant cannot be identified. Confidentiality will be maintained by removing all the identifiers and using the participant’s code number for data presentation.

The study team collecting data will be comprised of at least two individuals at each site, the First Nations research officer and clinician researcher. The clinician research lead, i.e., project coordinator, will monitor data collection and source documents during regular study meetings. Once a month, the project coordinator will verify the information that has been collected on the study database and monitor any missing data or errors in data entry. If regular errors in data entry or collection are noted, an action plan will be put in place.

All eligible participants will be included in the study. All researchers recruiting participants and collecting data will have a current Good Clinical Practice certification.

### Plans to promote participant retention and complete follow-up

Retention of participants for the 6–12-week follow-up phone call will be primarily achieved by ensuring the best contact number is provided by the parent at time of recruitment. In addition, if the parent does not answer the call, an SMS text will be sent to explain to the parent what the call is about. Lastly, the phone used to text and call the patient is from a visible (unblocked) number. Parents have suggested these steps to improve family engagement with follow up phone calls.

### Data management

Study data will predominantly come from two sources, (i) clinical care/medical records, and (ii) primary data collection, the latter of which will be entered directly into the study database. The researcher will utilise a REDCap (https://www.project-redcap.org/) database. REDCap is a secure, web-based, electronic data capture platform. The instance of REDCap that will be used is hosted on Telethon Kids Institute servers (within Western Australia), which are encrypted, behind multiple firewalls, contain intrusion detection systems in place, and are periodically backed-up (offsite, still within Western Australia) in line with institute policy. This permission restricted, web-based electronic data capture platform meets the recommendations of the Australian Clinical Data Interchange Consortium.

We will follow a formalised data management plan that details all clinical data to be regularly (fortnightly, minimum) entered on REDCap, with quality control reviews to be carried out monthly. A data audit will be completed by research staff to query missing entries. Unvalidated data will be excluded from analysis. Repeated data errors or omitted data will be investigated and resolved. The REDCap platform provides quality control functionality including data range and consistency checks. The use of REDCap ameliorates the risk of media obsolescence as data can be exported in a wide variety of formats.

Data entered into the REDCap database will be consistent with and substantiated by the patient’s medical record and other original source documentation. Source documentation will be legible, complete and attributable to an originator. The analysis of all collected data will be carried out by the study team. The study team directly collecting the data will be comprised of at least two individuals per site: the Aboriginal research officer and a second research officer. The project coordinator will monitor data collection and source documents during regular study meetings.

### Confidentiality

Participant identification will occur via daily audit of hospital admissions only accessed by a hospital respiratory clinician or paediatrician who would ordinarily have access to these data for clinical reasons, to protect patient confidentiality. The patient’s name and ward will be given to the research assistant who will visit the participant and provide information about the study. No data will be collected on the participant without informed consent. If a participant refuses consent, no data will be collected with exception that there was an admission where study consent was refused.

Once informed consent is obtained then standard data will be collected as per the protocol. Data will be collected from the participants, discharge summaries and the hospitals admissions database (e.g., iSoft).

Any written materials will be securely stored in a locked office with authorised access by swipe card only, in compliance with the Telethon Kids Institute Research Data Confidentiality policy. The data will be stored for a minimum of 15 years as per the NHMRC Guidelines.

**Plans for collection, laboratory evaluation and storage of biological specimens for genetic or molecular analysis in this trial/future use**

N/A

### Statistical methods for primary and secondary outcomes

Data will be exported from REDCap and processed into the form of one record per participant per visit. Basic descriptive statistics will be used to describe and characterise the data, including reporting means and standard deviations, medians and interquartile ranges, and counts and percentages, as appropriate.

The primary analysis for the study will be carried out using a mixed model framework. The primary outcome variable will be the absolute PC-QoL score; with the difference between the “nil-intervention” and the “post-intervention” groups being the value of interest. This value will be estimated by the coefficient of a 2-level fixed term from the model that indicates the “nil-intervention” and “post-intervention” groups of the study; it is this coefficient (and the accompanying two-sided 95% confidence interval) that will be reported as the main outcome of the study. This coefficient represents a ‘whole of program’ effect (difference between the nil-intervention and post-intervention groups). The model, at a minimum, will include both a fixed and random term for study site (to control for both the within site clustering and within person clustering, as we anticipate a number of participants may present in multiple observation periods) and will include the covariates of calendar week (to control for time and seasonal effects), sex (to control for risk differences by sex), and age (to control for risk differences by age). Adjusted analyses, such as that proposed, typically increases study power [28]. Model fit statistics and graphics will be examined. If a different model is deemed to provide a better fit than that described above, rationale to support this will be provided in the report.

Additional analysis of the primary outcome will be carried out, including a minimally adjusted model (using the same framework as described above, but with no covariates in the model, only within site clustering) and a non-adjusted model (using Student’s t-test). Basic comparisons for second outcome measures will utilise Chi-squared tests and Student’s t-tests (with comparison made between nil-intervention and health-information only data, health-information only and post-intervention, and nil-intervention and post-intervention data). In line with the primary outcome analysis, a mixed model framework will be used to examine the effect of prognostic factors of interest on binary (logistic) and count (Poisson) outcomes, where intervention-stage effects will be reported with 95% confidence intervals. A significance level of 0.05 will be used to determine statistical significance, though the focus on the analysis and reporting will be on the effect size estimates and their associated confidence intervals.

### Interim analyses

Interim analysis will occur for auditing of clinician adherence to the strategy. The audits will occur on 10 random discharges per month during a 6-month implementation period. The resultant summaries will be provided to HCPs at routine staff meetings (audit-and-feedback) to motivate ongoing change.

All other analysis will occur upon study completion. The decision to progress from the health information only control group to post-intervention group will be based on:

1. Meeting a minimum 12 PC-QoL tools per site or cluster of sites
2. The organizational functions being ready for strategy delivery.

### Methods for additional analyses (e.g. subgroup analyses)

Sub-group analysis may be run on different patient subgroups, such as (i) very young children aged 0–3 years without neurological involvement; (ii) children born pre-term; and (iii) children with neurological conditions, to explore risks of ongoing symptoms.

### Methods in analysis to handle protocol non-adherence and any statistical methods to handle missing data

A data audit will be completed by research staff to query missing entries. Unvalidated data will be excluded from analyses. Repeated data errors or omitted data will be investigated and resolved. Once a month, the project coordinator will verify the information that has been collected on the study database and monitor any missing data or errors in data entry. If regular errors in data entry or collection are noted, an action plan will be put in place.

### Plans to give access to the full protocol, participant level-data and statistical code

The protocol will be published and made available on request. The participant-level dataset will not be made public but will be available for non-public disclosure, if requested.

### Oversight and monitoring

#### Composition of the coordinating centre and governance structure

##### Steering Committee

The ACE Study Steering Committee will assume overall responsibility for the ACE study design and implementation. It will be comprised of the CPI, Clinical Lead, Collaborators, invited Site Principal Investigators (PIs), representatives of the ACE study community reference group, and invited representatives of key stakeholders. Steering Committee scope and membership are defined in The ACE Study Steering Committee Terms of Reference.

##### Executive Committee

The ACE Study Executive Committee will be comprised of key project members who will meet regularly to oversee the day-to-day management of ACE. It will be answerable to the Steering Committee but may make decisions on minor issues without requiring prior approval from the Steering Committee. It will ensure decisions made by the Steering Committee are communicated to, and implemented by, the Operations Group.

##### Operations group

The ACE Operations Group will comprise ACE project staff who will oversee study procedures, documentation and provide administrative support to the various governance groups. It will comprise the CPI, Clinical Lead, Project Manager, and other project staff. The ACE Operations group will be responsible for operationalising decisions of the Steering Committee.

##### Consumer reference group

Representatives from the Telethon Kids BREATH consumer reference group (CRG) will be invited to sit on the ACE Steering Committee. The CRG is made up of parents or other family members whose First Nations child/children have lived experience of chronic respiratory disease. Details of the reference group are found at the following site: https://www.telethonkids.org.au/our-research/chronic-and-severe-diseases/respiratory-health/BREATH/breath-consumer-reference-group-of-wa/

CRG members will have ongoing involvement in the design and implementation of the study. The CRG will be coordinated by the Operations Group.

**Composition of the data monitoring committee, its role and reporting structure**

N/A

**Adverse event reporting and harms**

N/A

**Frequency and plans for auditing trial conduct**

N/A

### Plans for communicating important protocol amendments to relevant parties (e.g., trial participants, ethical committees)

All protocol amendments must be documented in writing and submitted to all relevant ethics committees. The two primary committees are the Western Australian Aboriginal Health Ethics Committee and the Child and Adolescent Health Service Ethics Committee. As the University of Queensland Human Research Committee is part of the National Mutual agreement, all approved ethics documents will be submitted to the Queensland committee for endorsement. No changes will occur until amendments are approved by all relevant ethics committees.

All protocol deviations will be documented with specified reason. Any data, action taken, and consequences in patients and in the study will be documented. All documentation related to deviation will be stored in the investigator file.

### Dissemination plans

It is anticipated that the results of this research project will be published and/or presented in a variety of fora (i.e., presented at relevant medical congresses and published in peer reviewed journals). In any publication and/or presentation, information will be provided in such a way that participants cannot be identified. Confidentiality will be maintained by removing all identifiers and using participant codes for data presentation.

A two-page English plain summary (and First Nations languages as required) report will be disseminated to participating families at the conclusion of the study. The report will also be disseminated to the consumer reference groups, other AMSs who are in collaborations with the researchers (Kimberley Aboriginal Medical Service and Broome Aboriginal Medical Service) and the broader public in relevant communities. The researchers have a strong track record of communicating with all partners in their existing research projects with Aboriginal communities in the Kimberley. We have provided regular email updates, feedback to ethics committees in bi-annual reports, and meetings to disseminate information and seek feedback over the course of the project.

## Discussion

First Nations children hospitalised with ALRIs are particularly vulnerable to developing bronchiectasis. In Australia, studies have shown up to 19% of First Nations children have bronchiectasis within 24-months post-hospitalisation for pneumonia or bronchiolitis. The development of bronchiectasis can likely be prevented with effective, timely, and culturally secure treatment in many of these children. If children are screened for persistent wet cough one-month post-hospitalisation, and managed optimally if symptoms are present, then bronchiectasis can often be prevented. Yet, currently, there is no formal follow-up strategy for First Nations children hospitalised with ALRIs. This project addresses this gap and aims to improve the post-hospitalisation medical follow-up of First Nations children hospitalised with ALRIs that would thus lead to improved lung health.

### Rationale for our chosen approach

The implementation of evidence-informed interventions among First Nations populations has had limited success in the past. The underlying reasons include lack of partnered research with First Nations stakeholders, research approaches that are not culturally secure, and a focus on communities and health systems as separate. Our study uses a First Nations led approach that considers the community (families) and health systems, (i.e., the hospital and primary care centres) simultaneously [29].

Our project will employ mixed methods (qualitative and quantitative pre-post study) using Participatory Action Research (PAR) and implementation science approaches. The Consolidated Framework for Implementation Research (CFIR) [18] has been selected as the theoretical framework to identify barriers and facilitators (a determinant framework). The CFIR facilitates the identification of implementation barriers that can be mitigated to better navigate the individual and organisational changes required to promote successful implementation [30]. Within First Nations contexts, ensuring positive clinical practice change and patient attendance at medical follow-up is more likely to be effective if strategies are tailored to identified barriers and facilitators experienced by service providers and consumers [31]. In essence, effective implementation requires a First Nations-led PAR approach. A PAR approach ensures First Nations perspectives inform changes to optimise existing biomedical paradigms [32].

The study’s pseudorandomized stepped wedge, pre-post intervention design is appropriate because a randomised controlled trial design is not feasible in this First Nations context, where all stakeholders at both the clinical and community level see the need for changes to address the current gap in services. Further, the commencement of the study, and timing of the intervention will vary significantly between study sites/clusters due to the considerable amount of hurdles that has to be overcome at each study site before the study can commence, including challenges with accessing remote towns and communities where access can be impossible for extended periods due to flooding, COVID related restrictions to entry by non-essential workers imposed by government, local Aboriginal lore-time that varies from year to year, and can be imposed at short notice.

The stepwise analysis of change (e.g. clinician giving parent health information → discharge process promoting follow-up → parent help seeking at 1-month → quality of medical management → health related quality of life) resulting from the implementation process will, however, provide strong evidence of efficacy of the intervention. Multi-site replication may further strengthen study findings. Careful measurement of implementation outcomes will allow analysis of the relative efficacy of the intervention at different study sites.

A single-site study with a similar design was implemented successfully in a previous study [13]. The strategy implemented in the present study was co-designed with First Nations consumers and their primary care health services following extensive investigation of the barriers to recognising chronic wet cough among families and primary care clinicians in managing these symptoms according to clinical guidelines [13].

### Trial status

Approved by the Western Australian Aboriginal Health Ethics Committee on 4.06.2019 and by the Child and Adolescent Health Service Ethics Committee on 28.08.2019. Recruitment has not commenced.

## Supplementary Information

**Additional file 1.** Interview question guide parent.

**Additional file 2.** Semi-structured interview guide for healthcare provider. 

**Additional file 3.** Healthcare provider focus group guide.

**Additional file 4.** Semi-structured interview guide for healthcare provider.

**Additional file 5.** Semi-structured interview guide for healthcare providers.

**Additional file 6.** Medical record audit checklist form.

**Additional file 7.** Telephone follow-up at 6-12 weeks post discharge.

## Data Availability

All Chief investigators will have access to the final dataset. No additional data or material is available.

## Abbreviations

ALRI: Acute lower respiratory tract infection
KT: Knowledge translation
PAR: Participatory Action Research
AMS: Aboriginal Medical Service
PC-QoL: Parent-Proxy Cough related Quality of Life
PCH: Perth Children’s Hospital
CI: Chief Investigator
HCP: Healthcare provider
