# Supplementary material for: Implementation of a strategy to facilitate effective medical follow-up for Australian First Nations children hospitalised with lower respiratory tract infections: study protocol
Source: BMC Pulm Med. 2022 Mar 17;22:92. doi: 10.1186/s12890-022-01878-3 (PMC8929266; doi:10.1186/s12890-022-01878-3)
Supplement: Supplementary file 1 — Additional file 1. Interview question guide parent. [file 12890_2022_1878_MOESM1_ESM.docx]

Supplementary File 1

**Interview question guide parent/carer**

The clinician research officer and First Nations research officer first introduce themself and identify where they are from. One of the researchers explain about lung health and the link between acute and chronic disease using culturally secure tools and approach. Next the researcher explains that the hospital are going to implement strategies to help facilitate medical follow up for parents and the interview is to find out what families thing of the strategies and what barriers and facilitators to implementing the strategies might be.

The first questions are about how you feel about communication in the hospital.

1. How do you feel about the communication between medical staff and patients?
2. Did anyone explain the importance of follow – up care after being discharged from hospital?
3. How can we improve communication between medical staff and families?
4. Did any staff explain about lung health to you and how it may affect your child?

The next questions are about your experience and how you feel in the hospital

1. How was your experience being admitted to (name of hospital)?
2. Do you feel comfortable about asking for more information from the medical staff?
3. Do you feel that you were/are being listen to or understood?
4. Did you feel comfortable and safe at the hospital?
   - If no, did you feel that being Aboriginal or Torres Strait Islander person may have an impact on how staff acted towards you?

It is really important for parents to go to their local clinic a month after discharge so the local doctor can provide any care, especially if your child is still coughing.

1. What might make this task difficult? i.e. transportation, family issues, work issues. What might help overcome these issues?
2. What would help follow-up?
   - SMS texts reminder help?
   - Pamphlet with information and pictures?
   - Discharge letter for local doctor with clear instructions?
3. Do you know whether the discharge summaries reach your local doctor/clinic?
