# Supplementary material for: Implementation of a strategy to facilitate effective medical follow-up for Australian First Nations children hospitalised with lower respiratory tract infections: study protocol
Source: BMC Pulm Med. 2022 Mar 17;22:92. doi: 10.1186/s12890-022-01878-3 (PMC8929266; doi:10.1186/s12890-022-01878-3)
Supplement: Supplementary file 2 — Additional file 2. Semi-structured interview guide for healthcare provider. [file 12890_2022_1878_MOESM2_ESM.docx]

**Supplementary File 2: Semi-structured interview guide for healthcare provider (clinicians)**

Respiratory clinician to provide a brief overview of bronchiolitis/pneumonia and link to developing bronchiectasis and risk for First Nations children.

To ensure families seek medical help a month later, we know families need to be provided with culturally secure lung health information and told to follow up at one-month. We also know that the local doctors need clear instructions on how to manage the child and get the hospital discharge information.

We will be implementing a strategy at (name of hospital) to facilitate medical follow-up for First Nations children admitted with ALRIs. The new process requires the following: (explain map). Map adapted from Laird P et.al, 2021 Respiratory follow-up to improve outcomes for Aboriginal children: twelve key steps. *Lancet Reg Health West Pac*
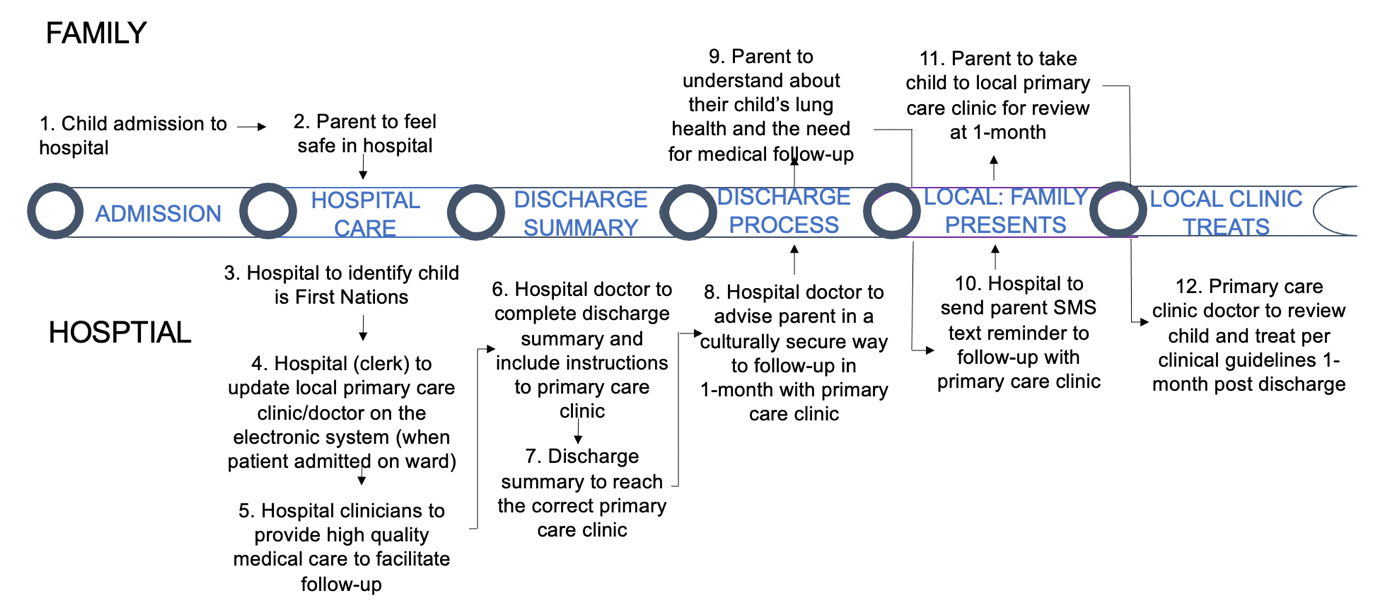


Questions

1. What do clinicians at the hospital need to know to implement the 12-key steps listed above as part of routine practice at the hospital?
2. How do we get buy-in at the hospital?
3. What are the facilitators? i.e., what do clinicians need – knowledge? time? protocols? Electronic reminders? Flow charts? Are there any other things might help?
4. What sorts of things might stop this process from happening? (barriers)
5. Can you tell us about any experiences you have had working with First Nations families at this hospital?

The next set of questions explore the cultural competence (i.e., knowledge, skills and understandings of staff

1. Do you feel confident to talk to families about lung health?
2. Do you feel like you have the skills to know how to engage with First Nations families?
3. Do you feel like you understand the families, and do you think the families understand your explanations? What would help you communicate with families?
4. Would you benefit from training in how to engage effectively with First Nations families? Would you prefer online or in-person and how do you think you could improve your skills in this area?
5. Do you know how to ask for interpreter services? Do you know about the service?

The following questions relate to improving First Nations family access to health care

1. What would help your families to adhere to the management?
2. What do you think the barriers are to families following up with their child?
3. What do you need to assist you to arrange follow up for a child?
4. Do discharge summaries get completed routinely and in timely way?
5. What are the barriers and facilitators to using a pre-populated template in the discharge summary with clear follow up instructions for local clinic?

Post-implementation

1. Did you find the system changes useful to facilitate follow-up?
   1. Do you think there are any ways to improve the follow-up process?
2. Did you find the resources to teach parents about lung health helpful?
   1. Can you suggest ways to improve these?
3. Did you find the training in culturally secure care helped with engaging with families?
   1. Can you suggest ways to improve these?
