# Supplementary material for: Implementation of a strategy to facilitate effective medical follow-up for Australian First Nations children hospitalised with lower respiratory tract infections: study protocol
Source: BMC Pulm Med. 2022 Mar 17;22:92. doi: 10.1186/s12890-022-01878-3 (PMC8929266; doi:10.1186/s12890-022-01878-3)
Supplement: Supplementary file 3 — Additional file 3. Healthcare provider focus group guide. [file 12890_2022_1878_MOESM3_ESM.docx]

**Supplementary File 3 Healthcare provider focus group guide**

Respiratory clinician to provide a brief overview of bronchiolitis/pneumonia and link to developing bronchiectasis and risk for First Nations children.

To ensure families seek medical help a month later, we know families need to be provided with culturally secure lung health information and told to follow up at one-month. We also know that the local doctors need clear instructions on how to manage the child and get the hospital discharge information. We will be implementing a strategy at (name of hospital) to facilitate medical follow-up for First Nations children admitted with ALRIs. The new process requires the following: (explain map) Map adapted from Laird P et.al, 2021 Respiratory follow-up to improve outcomes for Aboriginal children: twelve key steps. *Lancet Reg Health West Pac*


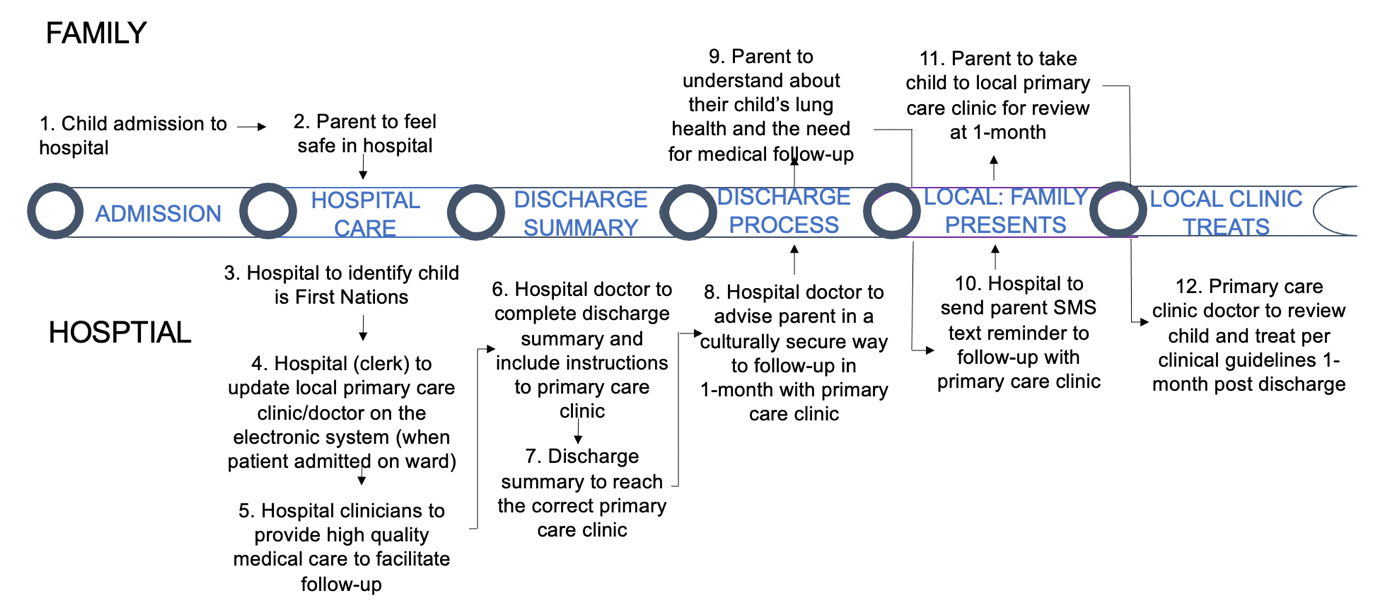


1. What do clinicians at the hospital need to know to do the above as part of routine practice at the hospital?
2. How do we get buy-in at the hospital?
3. What are the facilitators? i.e., what do clinicians need – knowledge? time? protocols? Electronic reminders? Flow charts? Or what other things might help?
4. What sorts of things might stop this process from happening? (barriers)

Post-implementation:

1. What worked well?
2. What can we do better?
