# Supplementary material for: Implementation of a strategy to facilitate effective medical follow-up for Australian First Nations children hospitalised with lower respiratory tract infections: study protocol
Source: BMC Pulm Med. 2022 Mar 17;22:92. doi: 10.1186/s12890-022-01878-3 (PMC8929266; doi:10.1186/s12890-022-01878-3)
Supplement: Supplementary file 4 — Additional file 4. Semi-structured interview guide for healthcare provider. [file 12890_2022_1878_MOESM4_ESM.docx]

**Supplementary File 4 Semi-structured interview guide for healthcare provider (administrative staff)**

1. When you started at hospital, during your induction, were you told to ask all patients about their Indigenous status?
2. Do you ask all families if they identify as being Aboriginal and or Torres Strait Islander?
   1. If no, why?
   2. What would you need in terms of skills and resources to do this?
   3. Did you know it was mandatory for all patients to be asked about their Indigenous status?
3. Do you ask about a patient’s local doctor?
   1. What do you do if they say they have no doctor?
   2. Do you then ask who their local clinic is?
4. Do you check what is the best contact number for the family and update it in the system?
5. When is the best time to get these kinds of details from the family?
6. Can you tell us about any experiences you have had working with First Nations families at this hospital?
7. Do you feel confident to talk to families?
8. Do you feel like you have the skills to know how to engage with First Nations families?
9. Do you feel like you understand the families?
10. What would help you communicate with families?
11. Do you know how to ask for Aboriginal interpreter services? Do you know about the service?

Post-implementation:

1. What worked well with the training?
2. What could be improved with the training?
3. Did the training help you ask about First Nations ethnicity?
4. Did the training help you ask about local clinic details?
