# Supplementary material for: Implementation of a strategy to facilitate effective medical follow-up for Australian First Nations children hospitalised with lower respiratory tract infections: study protocol
Source: BMC Pulm Med. 2022 Mar 17;22:92. doi: 10.1186/s12890-022-01878-3 (PMC8929266; doi:10.1186/s12890-022-01878-3)
Supplement: Supplementary file 5 — Additional file 5. Semi-structured interview guide for healthcare providers. [file 12890_2022_1878_MOESM5_ESM.docx]

**Supplementary File 5: Semi-structured interview guide for healthcare providers (local primary care clinics)**

Respiratory clinician to provide a brief overview of bronchiolitis/pneumonia and link to developing bronchiectasis and risk for First Nations children.

To ensure families seek medical help a month later, we know families need to be provided with culturally secure lung health information and told to follow up at one-month. We also know that the local doctors need clear instructions on how to manage the child and get the hospital discharge information.

We will be implementing a strategy at (name of hospital) to facilitate medical follow-up for First Nations children admitted with ALRIs. The new process requires the following: (explain map) Map adapted from Laird P et.al, 2021 Respiratory follow-up to improve outcomes for Aboriginal children: twelve key steps. *Lancet Reg Health West Pac*


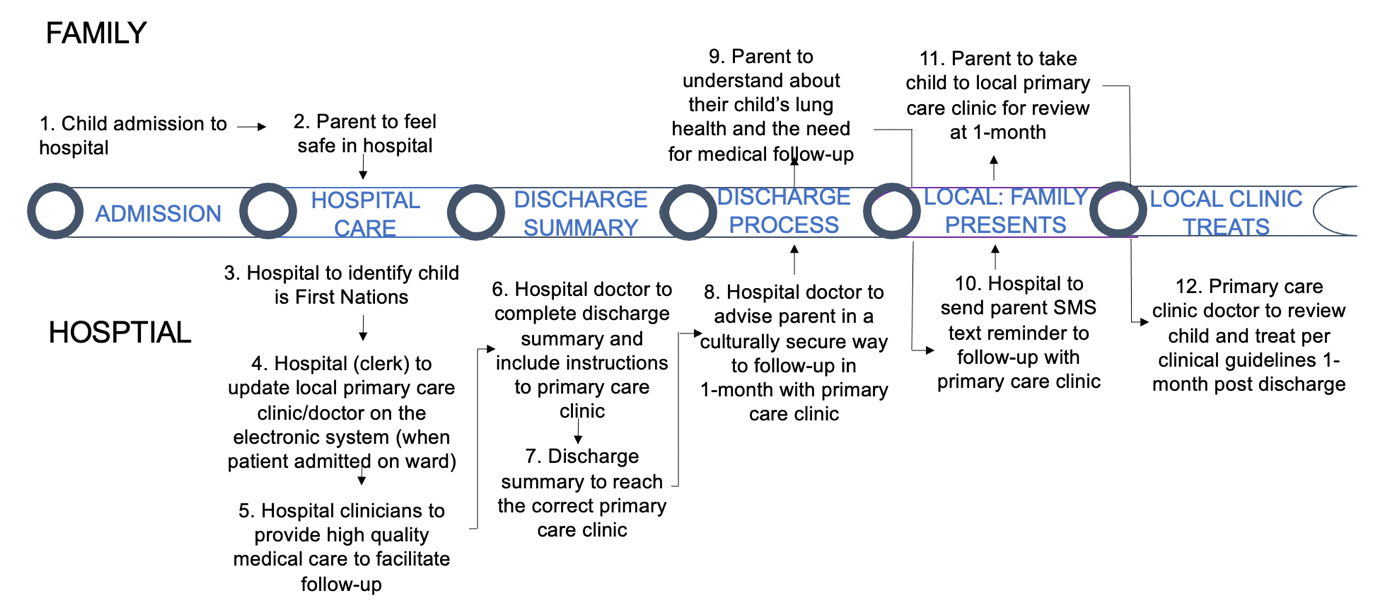


**Questions:**

1. What do clinicians at the clinic need to know to do the above to provide effective follow-up for children post-discharge at the local clinic?
2. How do we get buy-in at the clinic?
3. What are the facilitators? i.e., what do clinicians need – knowledge? time? protocols? Electronic reminders? Flow charts? Or what other things might help?
4. What sorts of things might stop this process from happening? (barriers)
5. What do you need to assist you to provide follow up for a child?
6. Do you get timely discharge summaries from the hospital?
   1. Do discharge summaries have follow up instructions clearly outlined?
   2. What would help facilitate receiving timely discharge summaries with clear instructions for local clinic?

**Post implementation**

1. Did discharge summaries arrive on time?
2. Was the discharge summary give clear instructions?
3. Did you know how to provide follow-up management for child?
4. What system changes would need to be made to improve the system?
5. Did parents present with their child and give a clear history?
