# Supplementary material for: Implementation of a strategy to facilitate effective medical follow-up for Australian First Nations children hospitalised with lower respiratory tract infections: study protocol
Source: BMC Pulm Med. 2022 Mar 17;22:92. doi: 10.1186/s12890-022-01878-3 (PMC8929266; doi:10.1186/s12890-022-01878-3)
Supplement: Supplementary file 6 — Additional file 6. Medical record audit checklist form. [file 12890_2022_1878_MOESM6_ESM.docx]

**Supplementary File 6:** **Medical record audit checklist form**

Date of audit: __________

Name of auditor: ________

| Patient name | UMRN | DOB | Visit Reason & Adm/Dis Date | Physician | Manag’t | DC summary completed by | Audit Checklist |
| --- | --- | --- | --- | --- | --- | --- | --- |
|  |  |  |  |  |  |  | 1. 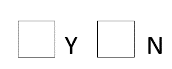 Disc. summary completed   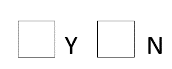   1. Doctor/clinic entered   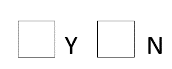   1. First Nations ethnicity noted   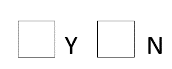   1. PT told to f/u in 4/52   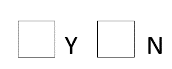   1. Doctor instructions included   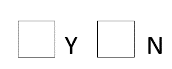   1. Specialist f/u |
