# Supplementary material for: Implementation of a strategy to facilitate effective medical follow-up for Australian First Nations children hospitalised with lower respiratory tract infections: study protocol
Source: BMC Pulm Med. 2022 Mar 17;22:92. doi: 10.1186/s12890-022-01878-3 (PMC8929266; doi:10.1186/s12890-022-01878-3)
Supplement: Supplementary file 7 — Additional file 7. Telephone follow-up at 6-12 weeks post discharge. [file 12890_2022_1878_MOESM7_ESM.docx]

**Supplementary File 7: Telephone follow-up at 6-12 weeks post discharge**

**CASE REPORT FORM (CRF)- SCREENING**

**Date of screening: Date of admission:**

**Please circle: Nil intervention group**

**Health information only group**

**Post-intervention group**

First name (interviewee):

Surname (interviewee):

Relationship with child:

First name (child):

Surname (child):

DOB:

Interviewer name:

1. Were you with (child’s name) during hospitalization? **YES / NO**

1a. (If **NO**), Can we get the contact details of who was present?

________________________________________________________________

1. When your child was in hospital, did the hospital staff (**other than the researchers**) explain (so you could understand) what was happening with your child and their lung health and how the sickness might impact your child?

**YES / NO / UNSURE / PREVIOUSLY EDUCATED**

2a. (If **YES**), who talked to you about lung health?

Doctor Nurse Physiotherapist Other Unsure Not Answered

1. Did someone suggest:

You take your child to doctor/clinic? **YES / NO / UNSURE**

You take your child to hospital? **YES / NO / UNSURE**

3a. (If  **YES**), When did they suggest you go?

Please circle:

No time given <1 week 1 week 2 weeks 3 weeks 4 weeks/ specialist clinic >1 month

Comments________________________________________________________

*For post-implementation group only

1. *Did you receive the text reminder at 1 month? **YES** **/**  **NO / UNSURE**

4a. *(If **YES**), Did the text help remind you to see a doctor? **YES** **/** **NO**

1. Did you take your child to the doctor post-discharge? **YES / NO**

5a. (If **YES**), When did you visit the doctor post-discharge?

Please circle:

Don’t remember <1 week 1 week 2 weeks 3 weeks 4 weeks/ specialist clinic >1 month

5b. (If **NO**), why?

________________________________________________________________

Any comments______________________________________________________

________________________________________________________________

1. What did the doctor do?

Please circle:

Prescribed antibiotics Nothing Other Gave medication but unsure which medication

6a. (If **Antibiotic**s), how long? Did you take them?

________________________________________________________________

6b. (If **OTHER**), what is it?_____________________________________________

Any comments______________________________________________________

1. Did your (child’s name) have a wet cough during admission? **YES / NO**

Any comments______________________________________________________

1. Did your (child’s name) have a wet cough during discharge? **YES / NO**

Any comments______________________________________________________

1. What happened to the cough?

________________________________________________________________

1. How long did the wet cough last?

________________________________________________________________
